# Analysis of risk factors affecting knee injury during yoga based on medical big data analysis

**DOI:** 10.1097/MD.0000000000043926

**Published:** 2025-08-22

**Authors:** Huiyan Li, Can Han, Lu Ma

**Affiliations:** a Sports and Health College, ABA Teachers University, Aba, Sichuan, China; b School of Physical, Xizang Minzu University, Xianyang, Shanxi, China; c School of Physical Education, Sichuan Normal University, Chengdu, Sichuan, China.

**Keywords:** knee joint injury, response, risk factors, yoga

## Abstract

The integration of big data systems into the medical field has diversified applications. Health and medical big data is a specific project of information technology application in the future development of medical field. Data analysis and functional configurations are implemented following comprehensive data collection. Yoga has a long history as a form of physical exercise. However, if practitioners do not pay attention to proper technique during practice, knee joint and other soft tissue injuries can easily occur. Based on this study through the big data collection to knee injuries, 89 yoga practitioners as the research object of this study, the other to select 100 cases of knee injury not occurring yoga practitioners as control group, compared to the general data, exercise habits, physical quality, such as information, using logistic regression model to cause a yoga practitioner of knee injury risk factors analysis; medical big data analysis revealed that high body mass index, low self-protection awareness, frequent forward-bending postures, insufficient sports medicine knowledge among yoga instructors, and incomplete curriculum design were independent risk factors for knee injury in yoga practitioners, and safety as the basic factor of choosing yoga was the protective factor. In addition to the risk factors affecting knee injury in yoga practitioners, the analysis of medical big data and the development of intervention measures on this basis may have certain clinical value in the prevention of this kind of injury. This confirms the medical data platform’s efficacy in developing yoga-related knee injury prevention strategies.

## 1. Introduction

At present, there are many researches related to data governance in China, but it is less applied in the medical field, generally applied in the fields of communications, finance, Internet, and so on. It can reflect the insufficiency of the information ability of most medical and health institutions. The development of healthcare big data in China remains in its early stages, particularly in terms of data governance. Moreover, high medical costs, many medical procedures, few medical resources, and small scope of knowledge popularization are still the shortcomings in the medical field in China.^[1,2]^ Therefore, it is of great significance to improve the prognosis of patients with different diseases by effectively utilizing medical data resources to mine the risk factors affecting different diseases and make targeted suggestions. As one of the oldest oriental fitness techniques, yoga is a mind–body exercise that integrates breathing, meditation, and posture.^[3]^ With the continuous development of the times, modern yoga includes flexibility, strength, and endurance exercises, which can not only strengthen the body and shape, but also dreg meridians, activate joints, and improve cardiopulmonary function.^[3]^ However, sports injuries associated with practice have not attracted public attention.^[4,5]^ This study aims to identify and analyze the risk factors contributing to knee injuries among yoga practitioners by leveraging medical big data analytics. Through a comparative analysis between yoga practitioners with and without knee injuries, the study utilizes a logistic regression model to explore variables such as body mass index (BMI), exercise habits, physical condition, and the role of yoga instruction quality. The ultimate goal is to provide evidence-based insights for injury prevention and inform safer yoga practice design through medical big data analysis.

## 2. Data sources and methods

### 2.1. Sources of information

This research through the use of medical technology, large data collected between January 2020 and June 2022, and 89 cases of knee injury occurred yoga practitioners as the research object, analysis of knee injury risk factors, and on the basis of the risk factors for making reasonable measures, contribute to the reduction of knee injury caused by yoga exercise. This study was approved by the Ethics Committee of ABA Teachers University, in March 2024. The data used in this study primarily originated from medical institutions and operational records generated by the platform itself, which can be divided into internal and external data sources. The internal data mainly includes the information system data of the municipal health Bureau, county and district health departments, secondary and above hospitals in the city, and primary medical institutions, while the external data is mainly the data shared and exchanged by other departments other than health departments.

### 2.2. Medical big data platform

**Figure 1.**
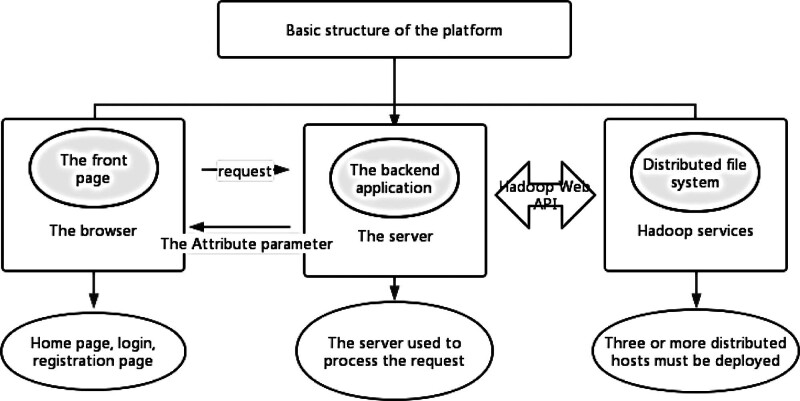
Architecture of the Medical Data Platform. This diagram illustrates the architecture of the medical data platform used in this study. The system is built on a distributed file system with a master node and data nodes, which manage storage, file paths, partitions, and backups. It includes Zookeeper for data management and system consistency, integrated with Hadoop. The platform uses a Tomcat server to provide secure, efficient static and dynamic services, ensuring easy deployment and resource optimization.

This research adopts the medical data platform mainly includes 6 aspects and 3 frame, frame structure as shown in Figure 1, the distributed file system, comprising master and data nodes, forms the platform’s foundational architecture, storage space for the entire file system, file name and path, the partition information, manage backup information and the storage location, and the external user access to the whole system of file resources, file operation control. In addition, Zookeeper, a distributed coordination service framework, solves the problem of data management and system consistency of the platform, and is also an important component of Hadoop. The main functions include: data publication and subscription, resource ID allocation, distributed system scheduling, distributed lock to ensure the consistency of different systems accessing the same resource, distributed work report, heartbeat detection, and distributed queue. The platform utilizes Tomcat server (Tomcat), selected for its low resource consumption and user-friendly deployment; support servlets and JSPS; it can provide both static resource access service and dynamic technology service. High security, external through the network can not directly access to the background code content; compatibility is good, many Web project software for the server to provide interface, and can be run directly in the software, very convenient and efficient.

**Figure 2.**
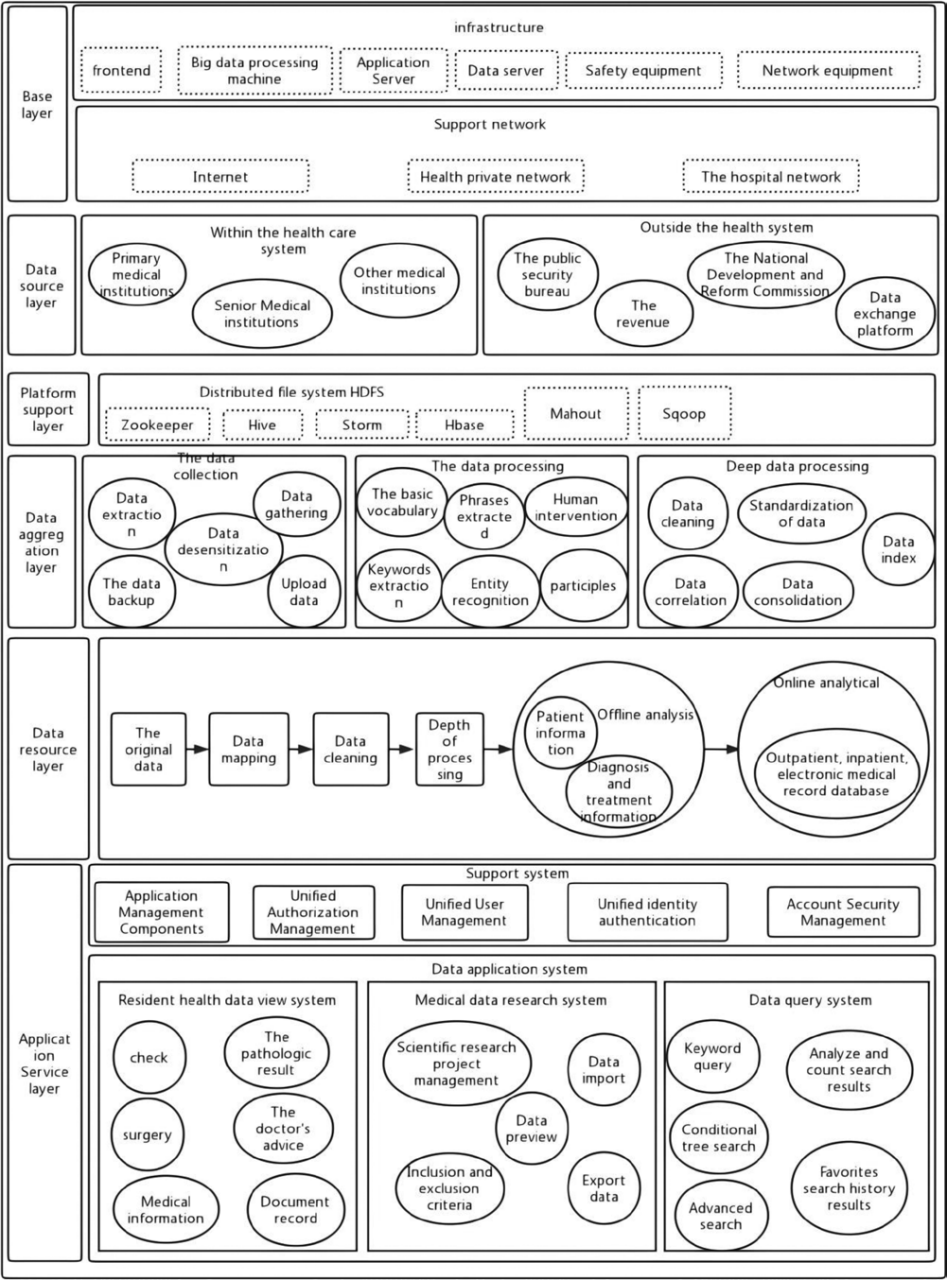
Layers of the Medical Data Platform. This figure illustrates the 6 key layers of the medical data platform architecture. The base layer provides the cloud platform, infrastructure, and support network for computing, processing, and storage resources. The data source layer collects data from both internal medical institutions and external sources from other departments. The platform support layer enables big data storage and computing, utilizing Hadoop technology for necessary computational resources. The data aggregation layer handles data collection, governance, and catalog management, processing the data into a unified catalog. The data resource layer consists of original, cleaned, standardized, and analyzed data, serving as the primary data resources for the platform. Finally, the application service layer provides various application services, such as disease mapping, medical data queries, and observation tools, enabling easier access and analysis of health-related data for residents.

The 6 layers are base layer, data source layer, platform support layer, data aggregation layer, data resource layer, and application service layer. Firstly, the base layer is mainly composed of cloud platform, infrastructure equipment and support network, which can provide computing, processing, storage, access, and other resources for the data in this study, and provide basic services such as computing power and network transmission for the application system. Secondly, the data source layer is also the main way to provide data sources for this study, including the data uploaded by medical institutions and the business data generated by the operation of the platform itself, which can be divided into internal and external data sources. Internal data included information from the municipal Health Bureau, district health departments, secondary and higher-level hospitals, and primary healthcare institutions, while the external data is mainly the data shared and exchanged by other departments other than health departments. Through the data source layer, the relevant data resources can be sorted out. In addition, the page number of the platform support layer is the main data computing platform, through which the storage and computing platform of hadoop technology can be provided for big data. The data aggregation layer includes data collection, governance, catalog management, and other items, which can further process the data of the data pool in a standard way, so as to form a unified data catalog. In addition, the data resource layer includes the collected original data, cleaned data, standardized data, and analysis data, which are also the main data resources supporting the database of the whole platform. Finally, the application service layer is the main platform that provides application functions for disease atlas, medical data query, medical data view observation, etc, so that residents can observe medical data more accurately, scientifically, and conveniently. The details of each layer of governance are shown in Figure 2.

### 2.3. Statistical processing (SPSS 25.0, IBM, Chicago, China)

#### 2.3.1. Normality testing

Normality tests were initially performed on the continuous variables using the Kolmogorov–Smirnov test. For variables that followed a normal distribution, descriptive statistics were presented as mean ± standard deviation. For variables that did not follow a normal distribution, non-parametric tests such as the Mann–Whitney *U* test were used for comparisons between groups.

#### 2.3.2. Hypothesis testing

Chi-square test: this was employed to examine categorical variables, determining whether there were significant differences between the 2 groups (yoga practitioners with knee injuries vs non-injured practitioners). For 2 × 2 contingency tables, Fisher exact test was used when expected counts were below 5.

Independent sample *t* test: this was used for comparing the means of continuous variables (e.g., BMI, age) between the 2 groups. Assumptions of normality and homogeneity of variance were checked using Levene test for equality of variances. If the variances were unequal, Welch *t* test was used instead.

#### 2.3.3. Multivariable logistic regression

To assess the relationship between multiple potential risk factors and knee injury in yoga practitioners, multivariable logistic regression analysis was performed. The following variables were considered: BMI, self-protection awareness, frequent postures (such as forward bending), knowledge of sports medicine among yoga instructors, and the completeness of the yoga curriculum.

Each independent variable’s significance was tested using Wald test with *P*-values (*P* < .05 considered statistically significant). Adjusted odds ratios were calculated to estimate the strength of association between each risk factor and knee injury.

#### 2.3.4. Confidence intervals

To assess the precision of the estimated odds ratios in the logistic regression model, 95% confidence intervals were calculated. Confidence intervals provide a range within which the true effect size is likely to fall, offering additional information on the uncertainty surrounding the estimated relationships.

### 2.4. Model validation

To ensure the validity of the logistic regression model, stepwise regression was used to select the most relevant variables for inclusion in the final model. Model fit was assessed using the Hosmer–Lemeshow test. This test evaluates whether the observed event rates match the predicted event rates across deciles of predicted probabilities. A *P*-value > .05 from the Hosmer–Lemeshow test indicates that the model fit the data adequately, meaning there was no significant difference between observed and expected values.

## 3. Results

### 3.1. The basic information and exercise characteristics of patients with knee injury and healthy group were compared

Data collected from the medical platform showed significant differences between yoga practitioners with and without knee injuries in terms of BMI, warm-up time, self-protection awareness, daily posture, elements of choosing yoga course, yoga instructor’s mastery of sports medicine and the systematic course, as shown in Table 1.

**Table 1:** Comparison of the basic information and exercise characteristics between the knee injury group and the healthy group.

	Knee injury group (n = 89)	Health groups (n = 100)	t/χ^2^	*P*
Age (yr)	23.24 ± 4.19	23.12 ± 4.86	0.181	.857
Gender			5.010	.125
Man	38 (42.70)	59 (59.00)		
Woman	51 (57.30)	41 (41.00)		
BMI (kg/m^2^)	22.92 ± 0.47	22.18 ± 0.62	9.158	<.001
Warm-up time (min)	6.72 ± 2.46	8.93 ± 4.67	-3.997	<.001
Sense of self-protection			11.274	<.001
Do not understand	42 (47.19)	11 (11.00)		
General understanding	37 (41.57)	67 (67.00)		
Very understanding	10 (11.24)	22 (22.00)		
Position the pose			5.643	<.001
Back bending position	11 (12.36)	23 (23.00)		
Forward-bending position	38 (42.70)	27 (27.00)		
Reverse position	16 (17.98)	26 (26.00)		
Inverted postures	24 (26.97)	24 (24.00)		
Choose the basics of yoga				
To achieve the purpose of fitness and plasticity	41 (46.07)	11 (11.00)		
It is ornamental	18 (20.22)	8 (8.00)		
More challenging	15 (16.85)	16 (16.00)		
Safety first	15 (16.85)	65 (65.00)		
Does the yoga instructor have a systematic knowledge of sports medicine			27.684	<.001
Y	35 (39.33)	77 (77.00)		
N	54 (60.67)	23 (23.00)		
Whether yoga classes are systematic			9.279	.002
Y	41 (46.07)	68 (68.00)		
N	48 (53.93)	32 (32.00)		

### 3.2. Multivariate analysis of high risk factors affecting knee joint injury in yoga practitioner

**Figure 3.**
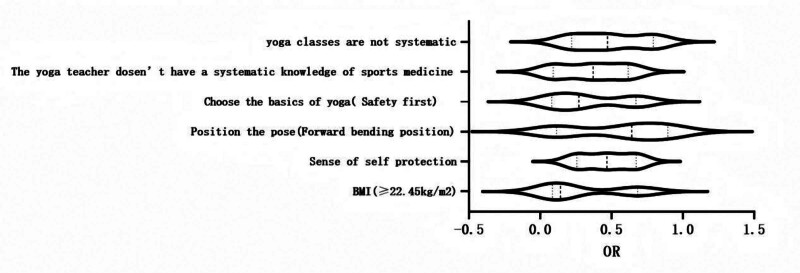
Multi-factor forest map.

Will be 2 groups have significant difference in the result, in the binary logistic regression model to analyze risk factors, the results showed higher BMI, ego to protect consciousness is poorer, often in a bending forward position, yoga instructor poor medical knowledge, and can result in yoga curriculum system incomplete athletes independent risk factors for knee injury, taking safety as the basic factor to choose yoga is the protective factor, see Table 2 and Figure 3 for details.

**Table 2:** Multivariate analysis.

	B	SE	Wald	*P*	OR	95% CI
BMI (≥22.45 kg/m^2^)	1.634	1.834	4.282	.002	0.142	0.085–0.683
Sense of self-protection	1.135	0.392	5.857	.023	0.467	0.256–0.673
Position the pose (forward-bending position)	3.385	1.686	3.183	.004	0.642	0.114–0.895
Choose the basics of yoga(safety first)	-1.752	0.328	3.495	.001	0.271	0.081–0.671
The yoga instructor dose not have a systematic knowledge of sports medicine	1.235	1.352	1.293	.001	0.371	0.091–0.617
Yoga classes are not systematic	4.381	2.482	2.592	<.001	0.472	0.219–0.793

CIs = confidence intervals, ORs = odds ratios.

## 4. Discussion

### 4.1. Application status of medical big data

The integration of big data system and medical field shows a trend of diversification. Health and medical big data represent a pivotal advancement in future healthcare technology. Data analysis and function setting are carried out based on data collection. These data require meticulous classification and management, broadly categorized into life/health data and medical data based on their applications.^[6,7]^ Medical data can be divided into 4 types: clinical data, health data, biological data, and clinical research. Life and health data can be divided into operation big data and public health big data. This includes 7 distinct types: industry governance data, management and decision-making data, public benefit data, and industrial development data.^[8]^ Operational data from the healthcare industry are stored on servers and processed using established digital models for classification, enabling direct decision-making support. The categorization of large data types and refine management can greatly reduced the speed of information retrieval, the user can according to their own requirements for information. As a result, the generation and application of big data will determine the future development direction of the health and medical industry to a large extent.^[9]^

In addition, according to the sources and functions of health and medical big data, it can be divided into 4 categories, namely, diagnosis and treatment assistance, health monitoring, public health, and targeted biomedicine.^[10]^ The sources of health and medical big data mainly include clinical data, scientific research data, and other data sets or databases in the real world. Health and medical big data originally came from manual records, and the increasingly in-depth combination of the Internet and medical treatment has promoted the explosive growth of data. In this process, problems in the development and application of health and medical big data have emerged, for example, network data are mostly hidden in complex layout patterns, from which valuable information needs to be obtained and its hidden value mined.^[11]^ While current technologies address some limitations, enhancing healthcare big data’s utility requires source-level improvements. Medical or research institutions need to have accurate understanding and reasonable positioning of data, and use appropriate technology at the right time to improve its practicality.^[12]^ Healthcare big data connects patients with similar diseases, enabling doctors to obtain information such as symptoms, side effects, hospitalization information, drug information, clinical report feedback, and drug efficacy, so as to provide patients with more accurate treatment plans.^[13]^

At present, there are many researches on data governance in China, but there are few applications in the medical field, which are generally applied in the fields of communications, finance, Internet, and so on. Although the relevant work has been carried out for many years and there are a large number of medical institutions, the medical data has been idle and has not been applied to generate further value, reflecting the lack of informatization ability of most medical and health institutions. The development of China’s health and medical big data is still in the early stage of data governance. Persistent challenges in China’s healthcare system include high costs, procedural complexities, limited resources, and insufficient health literacy.^[14,15]^ Therefore, it is of great significance to improve the prognosis of patients with different diseases by effectively utilizing medical data resources to mine the risk factors affecting different diseases and make targeted suggestions.

### 4.2. Status quo of yoga application

Yoga originated in ancient India and has a history of more than 5000 years. It is one of the oldest physical exercises in the East. However, as a pepresentatice of the oriental sports culture, Indian yoga is unique in the globalization context where the western sports culture is the mainstream, and with its unique charm, it has set off waves of “yoga fever” all over the world. With the spread of yoga, more and more studies have been conducted on the effects of yoga on the human body. If any sports item wants to develop with strong vitality, it must be backed by practical and powerful scientific research, and yoga as a new sports item is no exception.^[16,17]^ Yoga helps stabilize the autonomic ervous system, reduce stress, alleviate mental tension, and promote a positive emotional state. Some scholars have pointed out that yoga can make individuals feel happy and satisfied and contribute to their physical health.^[18]^ Another part of the scholars in the study of university physical education curriculum, found that the most significant effect of yoga on the reduction of anxiety and stress, and also have a positive effect on mood improve, their breathing method is put forward, the effect of deep breathing has stable mood, yoga is similar to progressive muscle relaxation training, have been shown to reduce anxiety, improve self concept, and reduce the pain of insomnia.^[19,20]^ However, sports injuries caused by yoga also need to be paid attention to. Although yoga is slow and gentle, most of the postures are forward bending, torsion, balance and back bending, so muscle strain and joint injury will occur if the exercise is not paid attention to. Therefore, to reduce unnecessary injuries, it is essential to use integrated medical big data system to systematically analyze the risk factors associated with yoga-related knee injuries and propose targeted preventive strategies.

### 4.3. Discussion of research results

Through the analysis of medical big data, this study identified several independent risk factors for knee injuries among yoga practitioners, including high BMI, poor self-protection awareness, frequent forward flexion, insufficient sports medicine knowledge among instructors, and an incomplete curriculum system. Additionally, safety-oriented course selection was found to be a protective factor. These findings provide valuable insights into the multifactorial nature of knee injuries in yoga, highlighting that knee injuries are a complex issue that requires consideration of various interconnected factors.

In contrast to existing research, many studies have focused on individual risk factors, such as BMI or specific yoga postures. For instance, Baum et al^[21]^ investigated the effects of varying stance widths during the triangle pose (Trikonasana) on lower extremity loading, finding that stance width significantly influenced joint load and injury risk. Their study aligns with our findings, which show that improper execution of specific postures, particularly forward flexion, increases knee joint stress. However, unlike their study, which focused on biomechanical factors, our research integrates multiple factors, such as BMI, self-protection awareness, and instructor knowledge, providing a more comprehensive understanding of knee injury risk in yoga.

Furthermore, our findings are consistent with Zhu et al,^[22]^ who identified a link between yoga practice and meniscus injury in Chinese women. While Zhu et al^[22]^ highlighted the risks of long-term yoga practice leading to meniscus injury despite weight reduction, our study emphasizes the importance of proper posture execution and instructor guidance. The role of instructors in ensuring safe practices is underscored in our study, as yoga instructors lacking sufficient knowledge of exercise physiology and sports medicine are a significant factor contributing to knee injuries.

In line with Wang et al,^[23]^ who found yoga beneficial for improving knee osteoarthritis symptoms, our study acknowledges the health benefits of yoga but also emphasizes that proper technique and instructor knowledge are paramount in preventing injuries. Wang et al^[23]^ found that yoga helps reduce pain and improve functionality in knee osteoarthritis patients, similar to the benefits observed in our study. However, our research takes a step further by incorporating a comprehensive view of safety and injury prevention, considering factors such as posture execution and instructor training.

Chan et al^[24]^ explored the relationship between sports participation and knee symptoms in young adults, highlighting that certain sports, such as yoga, are significantly associated with knee symptoms. The study found that participants who engaged in competitive sports, including yoga, were at a higher risk of experiencing knee symptoms compared to recreational players. This aligns with our findings that emphasize the importance of safe practices and mindful participation, particularly for individuals who practice yoga at a high intensity or competitive level. These results support the need for preventive measures, especially for individuals who may engage in yoga as a competitive sport.

The novelty of this study lies in its integration of multiple risk factors into a comprehensive data-driven analysis. Unlike previous studies that have focused on isolated factors, our research incorporates a holistic view of knee injury risks by considering BMI, posture execution, and instructor knowledge. This methodological innovation offers a more nuanced understanding of the complex factors that contribute to knee injuries in yoga, providing a broader and more detailed analysis than previous research, which often limited itself to examining individual aspects of yoga practice.

### 4.4. Suggestions for the yoga process

Based on the above risk factors, this study puts forward some suggestions for the protection of knee injury during yoga exercise. Firstly, the training arrangement of exercise should follow the change law of individual physical quality and physiological function. In addition, strengthening core strength training plays a role in stabilizing and supporting body posture and technical movements in sports. A strong core can stabilize the spine and pelvis, improve control and balance of the body, and enable the user to better maintain static poses, such as the boat pose series, and to freely transition between asanas, such as slant plate and plank, back bend and forward bend. Secondly, the body position arrangement of yoga class must comply with the scientific principles of movement human body, for example, twisting, large balance, large extension, and other large range of movements, should not be used as the starting posture, because this kind of movement range is large, the body needs to have a certain degree of adaptation. For yoga instructors, the protection of yoga sports injury must be included in the teaching objectives and given enough attention. Practitioners need to choose feasible goals according to their own needs, and recognize the goals, and persevere; reasonably grasp the range and intensity in the process of practice, do not over-demand themselves. Understand your own physical condition, joint tissue disease patients, patients with neurological disease is not recommended to practice yoga; students should use all their energy during the practice, focus on the breath and body in the moment, and focus on the practice. Other yoga venue construction to improve value, increase financial support, to keep the surface smooth and flat practice fields, safe and comfortable environment will help to achieve the desired results of practice yoga MATS, yoga brick and rope yoga practice items should also prepare enough, auxiliary equipment is of great help to the correct practice yoga postures, It can also reduce the damage caused by improper postures.

## 5. Limitations and future research

While this study offers valuable insights, it is not without limitations. One major limitation is the cross-sectional design, which limits our ability to draw causal conclusions. Future studies could benefit from longitudinal designs to observe the long-term effects of yoga practice on knee injuries and to evaluate the impact of specific interventions aimed at reducing risk. Additionally, incorporating more diverse demographic groups, including those with different cultural backgrounds and levels of experience, could further strengthen the generalizability of our findings.

Moreover, future research should explore the potential of machine learning techniques in predicting and preventing injuries based on various input variables, such as BMI, posture, and instructor knowledge. This approach, already applied in other fields (e.g., sports science), could offer even more precise and individualized risk assessments, enhancing injury prevention strategies for yoga practitioners.

## 6. Conclusion

This study identifies key risk factors for knee injuries in yoga practitioners, including high BMI, poor self-protection awareness, frequent forward bending, inadequate sports medicine knowledge among instructors, and an incomplete curriculum. Safety-focused course selection was found to be a protective factor. These findings highlight the importance of proper technique, instructor training, and a comprehensive curriculum to reduce knee injuries.

Utilizing medical big data provides valuable insights into injury risk factors and offers a strong foundation for developing targeted prevention strategies. Future research should focus on longitudinal studies and explore machine learning approaches for more accurate injury prediction and prevention. This study emphasizes the importance of data-driven strategies to enhance the safety of yoga practice.

## Author contributions

**Conceptualization:** Huiyan Li, Can Han, Lu Ma.

**Data curation:** Huiyan Li, Can Han.

**Formal analysis:** Huiyan Li, Can Han.

**Funding acquisition:** Huiyan Li, Lu Ma.

**Investigation:** Huiyan Li, Can Han, Lu Ma.

**Methodology:** Huiyan Li, Can Han.

**Validation:** Huiyan Li, Lu Ma.

**Visualization:** Can Han, Lu Ma.

**Writing – original draft:** Huiyan Li, Can Han, Lu Ma.

**Writing – review & editing:** Huiyan Li, Can Han.
